# Mutated LRRK2 induces a reactive phenotype and alters migration in human iPSC-derived pericyte-like cells

**DOI:** 10.1186/s12987-024-00592-y

**Published:** 2024-11-18

**Authors:** Sanni Peltonen, Tuuli-Maria Sonninen, Jonna Niskanen, Jari Koistinaho, Marika Ruponen, Šárka Lehtonen

**Affiliations:** 1 A.I. Virtanen Institute for Molecular Sciences, University of Eastern Finland, Kuopio, Finland; 2 Helsinki Institute of Life Science, University of Helsinki, Helsinki, Finland; 3 Drug Research Program, Division of Pharmacology and Pharmacotherapy, University of Helsinki, Helsinki, Finland; 4 School of Pharmacy, Faculty of Health Sciences, University of Eastern Finland, Kuopio, Finland; 5 Neuroscience Center, University of Helsinki, Helsinki, Finland

**Keywords:** Parkinson’s disease, LRRK2 G2019S, hiPSCs, Pericytes, Neuroinflammation, Migration

## Abstract

**Background:**

Pericytes play a crucial role in controlling inflammation and vascular functions in the central nervous system, which are disrupted in Parkinson’s disease (PD). Still, there is a lack of studies on the impact of pericytes on neurodegenerative diseases, and their involvement in the pathology of PD is unclear. Our objective was to investigate the molecular and functional differences between healthy pericytes and pericytes with the *LRRK2* G2019S mutation, which is one of the most common mutations associated with PD.

**Methods:**

Our study employed pericyte-like cells obtained from induced pluripotent stem cells produced from PD patients with the *LRRK2* G2019S mutation as well as from healthy individuals. We examined the gene expression profiles of the cells and analyzed how the alterations reflect on their functionality.

**Results:**

We have shown differences in the expression of genes related to inflammation and angiogenesis. Furthermore, we observe modified migration speed in PD pericyte-like cells as well as enhanced secretion of inflammatory mediators, such as soluble VCAM-1 and MCP-1, in these pericyte-like cells following exposure to proinflammatory stimuli.

**Conclusions:**

In summary, our findings support the notion that pericytes play a role in the inflammatory and vascular changes observed in PD. Further investigation of pericytes could provide valuable insight into understanding the pathogenesis of PD.

**Supplementary Information:**

The online version contains supplementary material available at 10.1186/s12987-024-00592-y.

## Background

Parkinson’s disease (PD) is the most common neurodegenerative motor disorder globally. Its incidence is rapidly increasing due to the aging of the population, and it is projected that by 2040, there will be 12 million people affected worldwide [1, 2]. PD is defined by the loss of dopaminergic neurons in the substantia nigra pars compacta and the formation of structures called Lewy bodies [3]. Most of the PD cases are sporadic. However, approximately 10% of the PD cases have a familial history, indicating a genetic component [4]. Several genes, including leucine-rich repeat kinase 2 (*LRRK2*), α-synuclein (*SNCA*), Pten-induced kinase 1 (*PINK1*), and parkin (*PARK2*) have been associated with familial forms of PD. Mutations in the *LRRK2* gene are also recognized as risk factors for sporadic cases. The familial PD caused by mutations in the *LRRK2* gene has clinical similarities to sporadic cases, with no apparent differences in the degeneration of dopaminergic neurons or appearance of Lewy bodies. Therefore, investigating mutations in the *LRRK2* gene might provide valuable insights into both familial and sporadic PD.

Research in PD has primarily focused on dopaminergic neuron degeneration and the pathological role of α-synuclein, a key component of Lewy bodies. Yet, less emphasis has been placed on the other aspects such as inflammation and vascular changes, despite evidence of chronic inflammation in the central nervous system (CNS) [5], indications of blood-brain barrier (BBB) disruption [6], and pathological angiogenesis in PD [7, 8]. Especially the research on pericytes, particularly in relation to PD and other neurodegenerative diseases, is very limited. Pericytes are a type of perivascular cells surrounding the microvessels. Due to their location around the vessels, they have an important role in vascular functions. In the CNS, they are needed for the formation and maintenance of BBB [9, 10]. Additionally, they regulate the process of angiogenesis [11]. Pericytes are also able to produce a wide variety of different inflammatory mediators, enabling their involvement in inflammatory signaling inside the CNS [12]. Thus far in PD research, it has been demonstrated that human primary pericytes are able to take up and degrade α-synuclein [13] and that α-synuclein affects vascular functions. Furthermore, it has been suggested that pericytes play a necessary role in the α-synuclein-induced hyperpermeability of the BBB [14]. These results are further supported by the study from Elabi et al. 2021, which showed that overexpression of α-synuclein triggers the activation of pericytes and BBB disruption [15]. However, it remains unknown whether the function of pericytes is modified in PD and the potential consequences of such alterations on PD pathology.

This study demonstrates the impact of the prevalent *LRRK2* mutation (G2019S) on both the transcriptome and functional levels of human induced pluripotent stem cell (hiPSC)- derived pericyte-like cells. Initially, we conducted a comparison between pericyte-like cells that were differentiated utilizing molecules TGFb3 or SB431542 to see how these cell profiles differ from each other in morphology and in protein and gene expression. Subsequently, we selected the protocol using the TGFb3 molecule for further differentiations and studied the disparities between hiPSC-derived pericyte-like cells that were differentiated from healthy lines and PD lines with the LRRK2 G2019S mutation.

## Materials and methods

### Culturing of hiPSCs

In this study, we used four healthy lines and three lines with G2019S mutation in the *LRRK2* gene (Table 1). The healthy line 4 and PD LRRK2 line 1 have been previously generated in the Stem Cell Core at the University of Eastern Finland and characterized [16]. The use of the patient-derived material has been approved by the Hospital District of Northern Savo, research Ethics committee (#42//2010 and #123//2016). Healthy lines 1–3 were commercial lines from Takara Bio, (Y00275, Y00305 and Y00325) and PD LRRK2 lines 2–3 were obtained from European Bank for induced pluripotent Stem Cells (EBiSC). Cell lines were maintained on Matrigel- (Corning) or Geltrex- (Gibco) coated plates in Essential 8 media (Gibco). When thawed, Y-27,632 ROCK inhibitor (Sigma) was used to enhance survival and attachment of the cells. Passaging of the cells was made with 0.5 mM EDTA (Invitrogen).

**Table 1:** The hiPSC lines used in the studies

Patient	Sex	Sample collection at age (years)	Genotype	Status	Reference	line identity
H1	F	20	Normal	Normal	TakaraBio	Y00270
H2	M	32	Normal	Normal	TakaraBio	Y00300
H3	M	32	Normal	Normal	TakaraBio	Y00320
H4	F		Normal	Normal	Holmqvist et al.	
PD1	M	64	LRRK2	Parkinson’s disease	Holmqvist et al.	
PD2	M	55–59	LRRK2	Parkinson’s disease	EBiSC	STBCi007-A
PD3	F	75–79	LRRK2	Parkinson’s disease	EBiSC	STBCi004-A

### Differentiation of hiPSC-derived pericyte-like cells

**Fig. 1:**
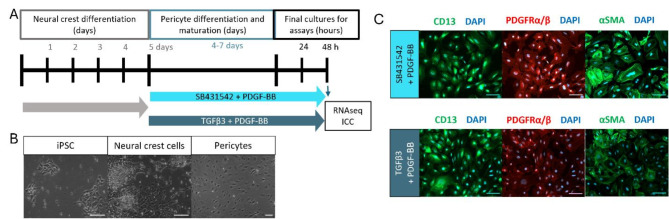
Differentiation and characterization of hiPSC-derived pericyte-like cells. (**A**) A schematic illustration of the differentiation of hiPSC-derived pericyte-like cells with PDGF-BB and SB431542 or TGFβ3. The hiPSCs are first differentiated to neural crest cells and then towards pericytes. (**B**) Representative bright field images of hiPSCs, neural crest cells and pericyte-like cells. (**C**) Representative fluorescent images of pericyte-like cells stained with CD13, PDGFRα/β and αSMA. Scale bar 100 μm

The protocol used in differentiation of pericyte-like cells is based on the protocol from Faal et al. (2019) (Timeline and images of different stages of differentiation, Fig. 1a, b). In the protocol, the cells were first differentiated into neural crest cells and then towards pericytes. For the differentiation of the neural crest cells, the hiPSCs were detached using Accutase (StemCell Technologies, Gibco) and plated 1.3-2 × 10^4^ cells/cm^2^ in E8 media with 10 µM Y-27,632 ROCK inhibitor on Matrigel coated dishes. Next day, the media was changed for the neural crest differentiation media (NCM: DMEM/F12 (Gibco), 2× B27 supplement (Gibco), 1× Glutamax (Gibco), 3 µM CHIR-99021 (Cayman) 0.5% BSA (VWR)) for 4 days. The media was changed daily and for the first two days Y-27,632 ROCK inhibitor was present to induce cell survival.

When the neural crest cells were differentiated, we tested two molecules (TGFβ3 and SB431542) to differentiate the pericytes. Even though SB431542 is an inhibitor of TGFβ signaling, both of these molecules have been shown to induce pericyte differentiation and have been used in protocols for pericyte differentiations [17, 18]. In differentiation of pericyte-like cells from neural crest cells, the neural crest cells were split to new Matrigel coated plates in pericyte media (PM: DMEM/F12, 1×B27, 1× Glutamax, 1× MEM NEAA (Gibco) with 5 µM ROCK inhibitor, 10 ng/ml PDGF-BB (Peprotech) and either 2 ng/ml TGFβ3 (Peprotech) or 10 µM SB431542 (TCI). Next day the media was changed to PM-media without ROCK inhibitor. PDGF-BB and TGFβ3 or SB431542 were kept in media until the cells were plated for the experiments, except for the ICC for the comparison of TGFβ3 and SB431542 and RNA sequencing in which the molecules were used until the samples were collected or fixed. The pericyte-like cells were split when needed on Matrigel coated plates and plated for the experiments after 4–7 days of culturing. The cell lines and amounts used in the experiments are listed in the tables (Tables 1 and 2).

**Table 2:** **Cell lines and amounts used in the experiments**

ICC	H3 (TGFβ3 vs. SB431542) H1, H2, H4, PD1, PD2 and PD3 (Healthy vs. PD) (from 1 differentiation batch)	- 2.5 × 10^4^/cm^2^
RNA-sequencing and qPCR	H1, H2, H3, PD1, PD2 and PD3 (from 2 differentiation batches)	2.5 × 10^4^/cm^2^
Cytometric bead array	H1, H2, H3, PD1, PD2 and PD3 (From 3 differentiation batches)	7.5 × 10^4^/cm^2^ (E-plate)
Scratch wound	H1, H2, H3, H4, PD1, PD2 and PD3 (From 3 differentiation batches)	5 × 10^4^/cm^2^
α-synuclein ELISA	H1, H2, H3, PD1 and PD3 (From 2 differentiation batches)	2.5 × 10^4^/cm^2^
Permeability tests	ECs: H1 (From 2 differentiation batch) Pericytes: H1, H2, H3, H4, PD1, PD2 and PD3 (From 3 differentiation batches)	1.35 × 10^5^/cm^2^ 6.7 × 10^4^/cm^2^

### Differentiation of hiPSC-derived endothelial cells

HiPSC-derived endothelial cells (hiPSC-ECs) were differentiated based on the protocol from Harding et al. (2017) [19]. Shortly, the hiPSCs were detached with accutase and plated 15 × 10^3^ cells/cm^2^ in E8 media with Y-27,632 ROCK inhibitor. Next day the media was changed for StemDiff APEL2 medium (StemCell Technologies) with 6 µM CHIR for two days, after which the media was changed for StemDiff APEL2 medium with 25 ng/ml BMP4 (Peprotech), 10 ng/ml FGFb (Peprotech) and 50 ng/ml VEGF (Peprotech) for 3 days. After differentiation, VE-cadherin positive cells were sorted by MACS columns using CD144(VE-cadherin) microbeads (Miltenyi). The hiPSC-ECs were cultured in ECGM MV2 (PromoCell).

### Immunocytochemistry (ICC)

The cells were fixed either with 4% formaldehyde (VWR) solution in PBS (EuroClone) at RT or MeOH (VWR) at 4 °C for 15–20 min and then washed with PBS. Before staining, the cells were permeabilized with 0.2% Triton-X 100 (Sigma) for 20 min and blocked with 5% horse serum (Gibco) in PBS at RT for 1 h. The cells were then incubated in 5% horse serum with primary antibodies overnight at 4 °C. Next day the cells were washed and incubated in a secondary antibody solution made in PBS or 5% horse serum for 1 h. Cells were washed again, and nuclei were stained with DAPI (0.5 µg/ml, Thermo Fisher). The cells were imaged with Zen Imager AX10. Used antibodies are listed in supplementary (Table S1).

### RNA sequencing

For RNA sequencing, the RNA was collected directly after cultures. The cells were lysed on ice (Buffer RLT with β-mercaptoethanol 10 µl/ml) and the RNA was extracted using RNeasy Mini Kit (Qiagen) with DNase I digestion using RNase-Free DNase Set (Qiagen). The RNA concentration was measured with DS-11 FX Spectrophotometer/Fluorometer (DeNovix), and 2 µg of the RNA-samples were sent to Azenta Life Sciences for RNA sequencing. Library preparation included the following steps: ribosomal RNA depletion, RNA fragmentation and random priming, cDNA synthesis, end repair, 5’ phosphorylation and dA tailing, and finally adaptor ligation and PCR enrichment. The sequencing was made with an Illumina NovaSeq 6000 instrument, PE 2 × 150. The reads were trimmed with trimmomatic v.0.36 to remove possible adapter sequences and poor-quality reads and trimmed reads were mapped to the Homo sapiens GRCh38 reference genome using STAR aligner v.2.5.2b.

Unique gene hit counts were calculated with featureCounts from the Subread package v.1.5.2. The gene hit counts were used for downstream differential expression analysis (DESeq2) which was utilized to compare the expressions between the sample groups, p-values and log2 fold changes were generated with Wald test. Genes with an adjusted p-value < 0.05 and an absolute log2 fold change > 1 were called differentially expressed genes (DEGs) for each comparison. For the comparison of the differentiation molecules, pathway enrichment analysis (Kyoto Encyclopedia of genes and genomes, KEGG, and gene ontology biological processes, GO BP) was made on DEGs but as in comparison between healthy and PD lines there were only 43 DEGs, so we used genes with a p-value < 0.05 instead of only the genes with adjusted p-value < 0.05. The pathway analysis was performed with Enrichr.

### RT-qPCR

For real time qPCR, the RNA was extracted as for the RNA sequencing from the fresh samples. The RNA was converted to cDNA with Maxima Reverse Transcriptase (ThermoFisher). For RT-qPCR we used Maxima Probe/ROX qPCR master mix (ThermoFisher) and Taqman assays (Table S2) to quantify relative expressions of genes. The RT-qPCR was conducted with Step One Plus (Applied Biosciences). The Ct values were normalized to the mean Ct value of house-keeping gene β-actin and the relative expression values were presented as a fold change.

### Permeability assay

The permeability tests were made for the monocultures of hiPSC-ECs and co-cultures of hiPSC-ECs and pericyte-like cells to see whether pericyte-like cells increase the tightness of the EC monolayer. The ECs were plated on the apical side of Matrigel coated 24-well TC inserts (Sarstedt, 3 μm pore size). As a control, we used inserts with only Matrigel coating without cells as well as empty inserts without any coating. Next day the inserts were flipped upside down and pericyte-like cells were plated in 1 mg/ml Matrigel in PM-media on basolateral side of the inserts. After plating, cells were incubated at 37 °C for 30 min, after which the inserts were flipped back to the well plates and the medium was changed for co-culture medium (1:1 DMEM/F12 and human endothelial SFM (Gibco), supplemented with 0.5×B27, 0.5× Glutamax, 0.5× MEM NEAA, 0.5× N2 (Gibco) and 5 ng/ml FGF). For inserts without pericyte-like cells, 1 mg/ml Matrigel solution in media without cells was plated.

The permeability was measured with 4 kDa FITC dextran and 20 kDa TRITC dextran (Merck), after 4 days of co-cultures. 150 µl of Dextran solution (0.5 mg/ml of FITC- and TRITC dextrans) was added to the apical side and 800 µl medium for the basolateral side. Samples of 80 µl were collected from basolateral side at 20-, 40-, 60- and 90-min time points, and the removed media was replaced with fresh media. At 90 min, samples were also taken from the apical side of the insert. If cells were exposed to IL-1β, the cultures were first cultured for 3 days, after which media was changed to co-culture medium with IL-1β (10 ng/ml) and after 24 h exposure, the permeability tests were made.

The fluorescent values were measured immediately after the permeability tests with a Victor2 multilabel plate reader (PerkinElmer). The background was reduced from the fluorescent values, and corrected fluorescent values were calculated to compensate for replacing the taken samples with fresh media using formula RFU_t, c_= RFU_t_ + (RFU_t−1_× V_s_/V_bas_), where RFU_t, c_ is the corrected signal, RFU_t_ fluorescence at a specific time point, RFU_t−1_fluorescence at the previous time point, V_s_ volume of the taken sample and V_bas_ volume of the media in basolateral side of the insert. The permeabilities were calculated as apparent permeability (Papp) -values from the corrected fluorescent values. Accumulated amounts of dextran on the basolateral side of the inserts were calculated using standards, after which the amounts were plotted against time to get dextran flux (linear regression) across the barrier. The Papp value was calculated using a formula $\:Papp=\frac{dQ}{dt}\times\:\frac{1}{A\times\:{C}_{0}}$, where dQ/dt is dextran flux across the barrier, A is the area of the insert membrane and C_0_ is the initial dextran concentration (µg/cm^3^).

### Cytometric bead array

The media samples from unexposed and IL-1β (Peprotech, 10 ng/ml) -exposed pericyte-like cells were collected on ice and stored at -70 °C. To determine the cytokine secretion levels, the cytometric bead array (CBA) was performed using Human Soluble Protein Master buffer Kit (BD) utilizing CytoflexS (Becman Coultier) for sample analysis. A minimum of 300 events per cytokine were recorded from the samples. The gained data was analyzed with FCAP Array v 2.0.2. (SoftFlow, Hungary). Regression analysis from standard concentrations was used to calculate the cytokine concentrations. Capture beads used were human soluble CD106 (VCAM-1), human IL-6, human soluble CD54 (ICAM-1) and human MCP-1.

### Scratch wound assay

The wound was made for the pericyte-like cells plated on a Matrigel-coated Imagelock 96-well plate (Sartorius, BA-04856) with IncuCyte WoundMaker and the detached cells were washed out with PBS. After washing, the media as a control, media with PDGF-BB (10 ng/ml) or IL-1β (10 ng/ml) was added for the wells. Images were taken once in an hour for 24 h in IncuCyte S3/SX3 live-cell-imaging (Sartorius) with 10x objective. The images were analyzed with integrated cell migration module, and migration speed was analyzed from relative wound density using time points of 3 h and 18 h with formula, Migration speed=∆ relative wound density % / ∆ time.

### α-synuclein ELISA

To measure endogenous α-synuclein in the pericyte-like cells, a human α-Synuclein ELISA kit was used (Invitrogen). The cells were lysed with cell extraction buffer (Invitrogen) and diluted in reagent diluent (1:10). The absorbance at 450 nm was measured with a VICTOR2 (Perkin Elmer) multilabel plate reader.

### Data analysis

We analyzed the data using GraphPad Prism. When analyzing RT-qPCR results, either unpaired t-test or one-way ANOVA and multiple comparison with Bonferroni correction were used. For the results from permeability tests, CBA and migration assay, we used a two-way ANOVA with Bonferroni’s or Tukey’s multiple comparison test. The used significance levels were *p < 0.05; **p < 0.01 and *** *p* < 0.001. To detect outliers, we used GraphPad Grubbs’ test. The transcriptomic data was visualized with a free online platform: https://www.bioinformatics.com.cn/en.

## Results

### Differentiation and characterization of hiPSC-derived pericyte-like cells

For deriving the pericyte-like cells from neural crest cells, we tested two different molecules, TGFβ3 and TGFβ signaling inhibitor SB431542, both of which are used in directing the differentiation towards pericytes. Furthermore, PDGF-BB was also used to induce differentiation of the cells towards pericytes, alongside these molecules. Both TGFβ3 and SB431542 generated cells with similar morphology and expression of commonly used pericyte markers CD13, PDGFRα/β and αSMA at the protein level. There were no visible differences in the levels of expression (Fig. 1c).

### TGFβ3 induces higher expression of pericyte associated genes compared to SB431542

**Fig. 2:**
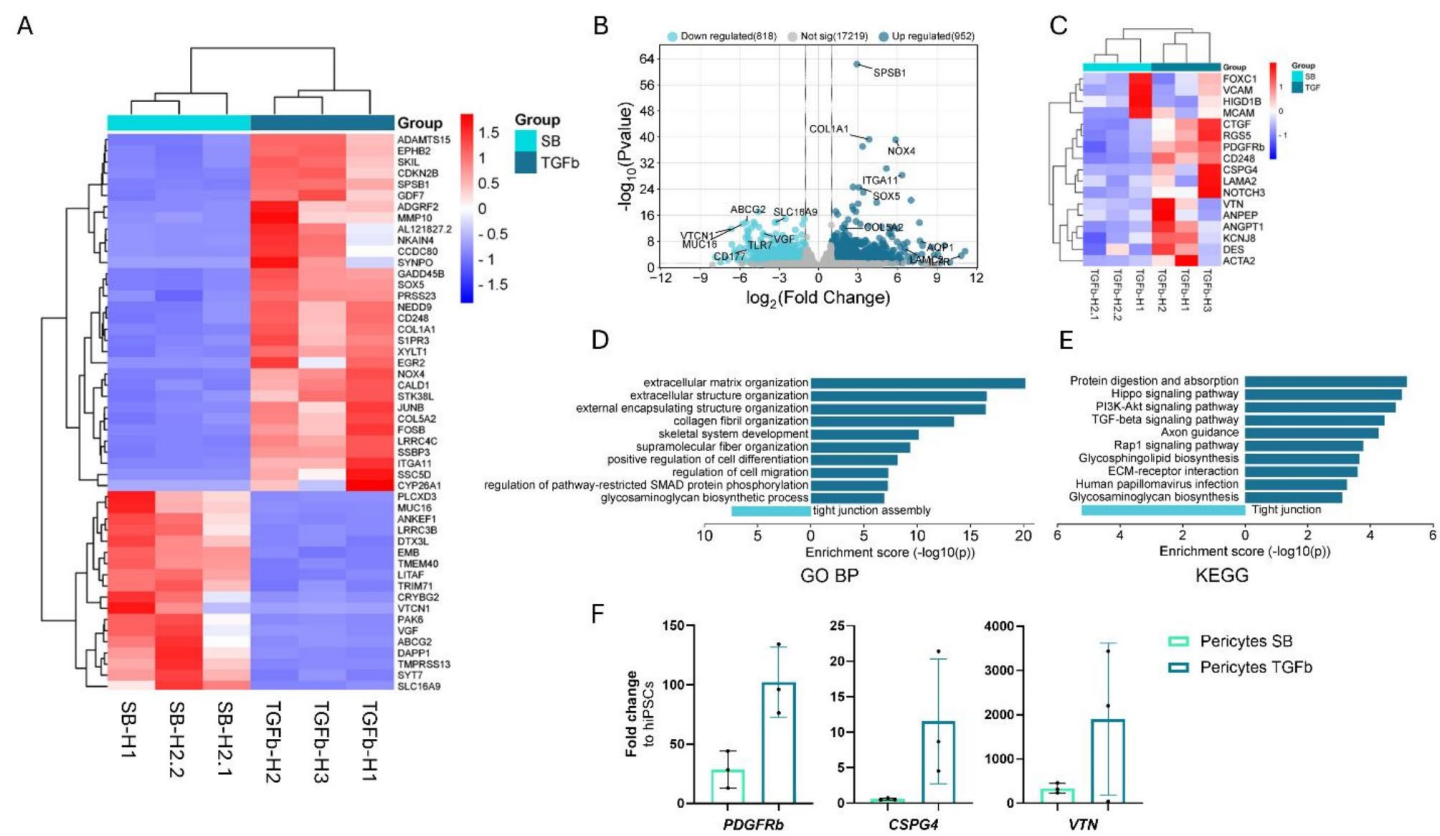
RNA expression analysis of hiPSC-derived pericyte-like cells differentiated with SB431542 and TGFβ3. (**A**) Cluster heatmap of the top 50 significant differentially expressed genes between SB431542 and TGFβ3 differentiated pericyte-like cells based on Padj value (< 0.05) and absolute log2 Fold > 1 (**B**) Volcano plot of up- and downregulated DEGs between SB431542 and TGFβ3 differentiated cells. (**C**) Cluster heatmap of pericyte associated genes between SB431542 and TGFβ3 differentiated cells. (**D**) GO pathway analysis of up- and downregulated genes in TGFβ3 differentiated cells compared to SB431542 differentiated cells. (**E**) KEGG pathway analysis of up and down regulated genes in TGFβ3 differentiated pericyte-like cells compared to SB431542 differentiated cells. (**F**) Relative gene expression of pericyte associated genes PDGFRb, CSPG4, and VTN compared to expression in hiPSCs, measured with RT-qPCR. Pericytes SB431542 (*n* = 3), Pericytes TGFβ3 (*n* = 3). KEGG, the Kyoto Encyclopedia of Genes and Genomes; GO BP, gene ontology biological processes. n = number of replicates

In order to elucidate the differences between TGFβ3 and SB431542 differentiated pericyte-like cells, RNA sequencing was performed. An adjusted p-value of < 0.05 was used to identify the differentially expressed genes (DEGs). The findings revealed that 818 genes were downregulated, and 952 genes were upregulated in TGFβ3 differentiated pericyte-like cells in comparison to SB431542 differentiated cells (Fig. 2b). Next, we compared the expression of genes associated with pericytes between pericyte-like cells differentiated with TGFβ3 and SB431542. Our analysis showed that a number of these genes (*ACTA2*,* ANGPT1*,* ANPEP*,* CD248*,* CSPG4*,* CTGF*,* DES*,* KCNJ8*,* LAMA2*,* NOTCH3*,* PDGFRβ*,* RGS5*, *and VTN*) were upregulated in TGFβ3 differentiated cells (Fig. 2c). The altered gene expression levels were confirmed with qPCR, showing the increased expression of *PDGFRβ*, *CSPG4*, and *VTN* (Fig. 2f) even though the differences were not statistically significant. Among the top upregulated genes, we also identified several genes encoding extracellular matrix proteins (*COL1A1*,* COL5A2*, *and LAMC2*). To gain a better understanding of the distinction between TGFβ3 and SB431542 differentiated cells, we utilized EnrichR to evaluate the DEGs and identify the modified pathways. The Gene ontology biological processes (GO BP) analysis revealed that processes related to extracellular matrix were upregulated in TGFβ3 differentiated pericyte-like cells, along with regulation of cell migration (Fig. 2d). On the contrary, processes related to tight junctions were downregulated. The downregulation of tight junctions was also identified in the KEGG pathway analysis. Furthermore, in the KEGG pathway, protein digestion and absorption, as well as ECM-receptor interaction, were increased (Fig. 2e). When we compared the pathways of differentiated pericytes compared to brain pericyte related pathways (based on CellMarker_Augmented_2021 data set including 98 pericyte genes) (Fig. S1) we noticed that many of the pericyte related pathways were upregulated in TGFβ3 differentiated cells. In summary, based on the higher expression of pericyte markers and upregulation of pericyte associated pathways, we have decided to proceed with TGFβ3 differentiated pericyte-like cells for further investigations.

### Human iPSC derived pericyte-like cells demonstrate pericyte-like properties

**Fig. 3:**
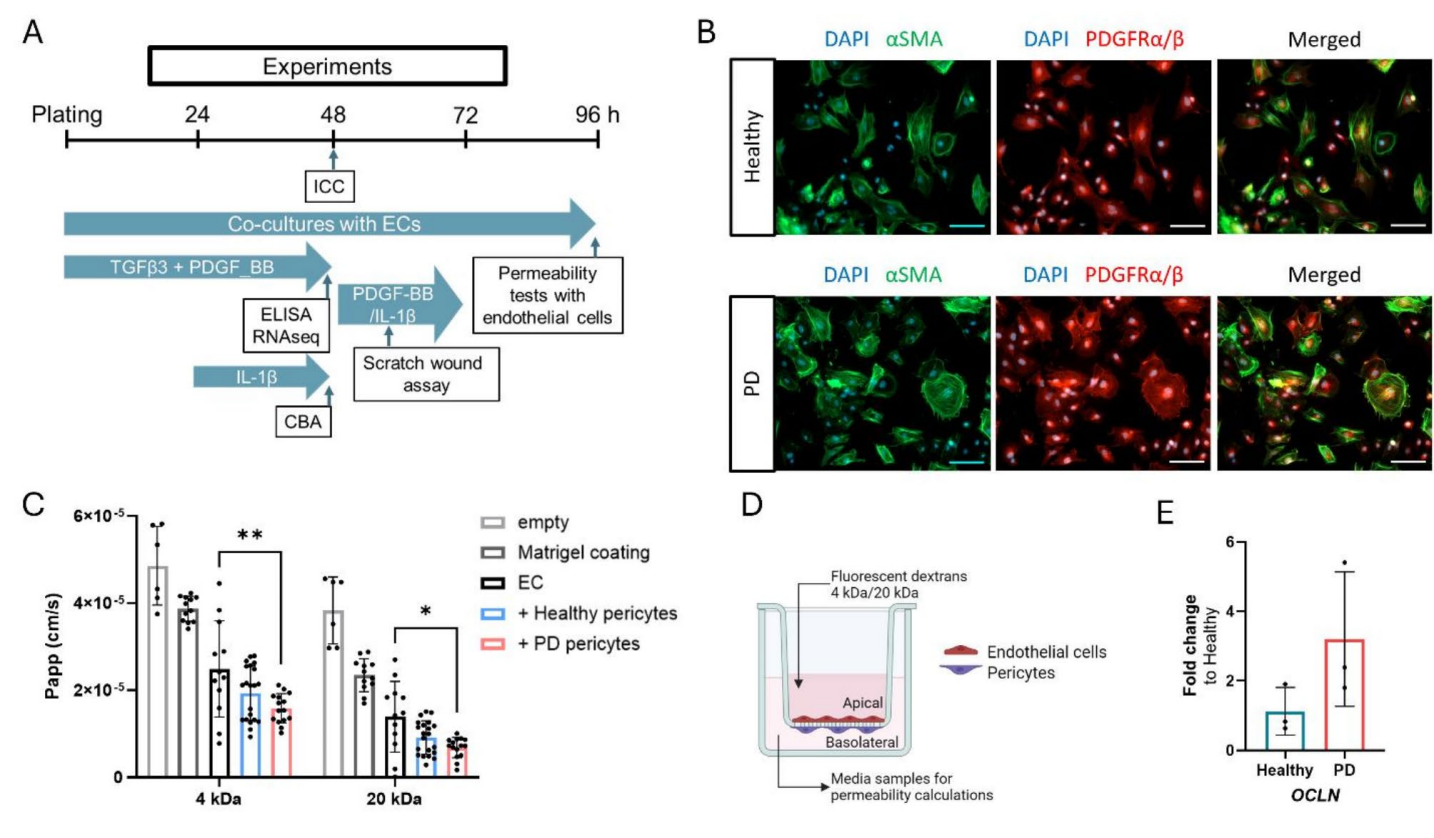
Differentiation and characterization of hiPSC-derived pericyte-like cells from healthy and PD lines. (**A**) A schematic illustration of the experiments timeline after plating. (**B**) Representative fluorescent images of pericyte-like cells stained with αSMA and PDGFRα/β. Nuclei stained with DAPI. Scale bar 100 μm.(**C**) Permeability (Papp) of 4 kDa and 20 kDa dextrans. Empty control (*n* = 6), Control with Matrigel coating (*n* = 12), hiPSC-derived ECs (*n* = 12), hiPSC-derived ECs + healthy pericytes (*n* = 21), hiPSC-derived ECs + PD pericytes (*n* = 15) (+/-SD). Two-way ANOVA with Tukey´s multiple comparison test. (**D**) Illustration of co-culture model of pericyte-like cells and ECs used for permeability tests. Created in BioRender, https://BioRender.com/x04o046. (**E**) Relative expression of tight junction gene OCLN in pericyte-like cells compared to healthy lines (*n* = 3) (+/-SD). Unpaired t-test. n = number of replicates, p*<0.05, p**<0.01

The pericyte-like cells, which were generated using TGFβ3, were now further characterized (Fig. 3a, b,c). This included the use of hiPSC lines obtained from both healthy donors and PD patients carrying the mutation in the *LRRK2* gene. The expression levels of pericyte markers, like alpha smooth muscle actin (αSMA) and PRGFRα/β, and morphology of the cells, were consistent across all lines (Fig. 3b, Fig. S2). Since we are examining the mutation in the *LRRK2* gene, we also checked the expression of *LRRK2* gene in our cells. *LRRK2* was detected in both healthy and PD pericyte-like cells with no significant differences in expression level (Fig. S4). Furthermore, as one of the characteristics in PD is the accumulation of α-synuclein in the CNS, we measured gene expression of *SNCA* with RT-qPCR as well as intracellular α-synuclein levels from the cells with ELISA. The mRNA expression of *SNCA* was seen in the cells without differences between studied lines (Fig. S4). However, despite the presence of *SNCA* expression in the cells, intracellular α-synuclein was not detected in either healthy or PD lines.

To investigate how pericyte-like cells affect barrier formation, we cultured pericytes with hiPSC-derived ECs on cell culture inserts. In this model, we plated the ECs on the apical side of the membrane, while pericytes were placed on the basolateral side. We then tested barrier formation with 4 kDa and 20 kDa dextrans (Fig. 3c, d). The dextrans permeated across the cell layers in a size selective manner. Surprisingly, the co-culture of ECs with PD pericytes produced a tighter barrier as calculated by apparent permeability (Papp) -values, when compared to EC monocultures. However, there was no significant difference in permeabilities between co-cultures of ECs with healthy and PD pericytes. Both monocultures and co-cultures resulted in significant reduction in permeability compared to Matrigel coated or empty inserts without any coating. After observing that the PD pericyte-like cells, when co-cultured with ECs, enhanced barrier formation, we proceeded to examine the expression of tight junction genes in these pericyte-like cells. We found that the expression of *OCLN* gene was increased in PD lines, but the difference was not statistically significant (Fig. 3e).

### LRRK2 G2019S mutation affects expression of angiogenesis inflammation and extracellular matrix organization associated genes

**Fig. 4:**
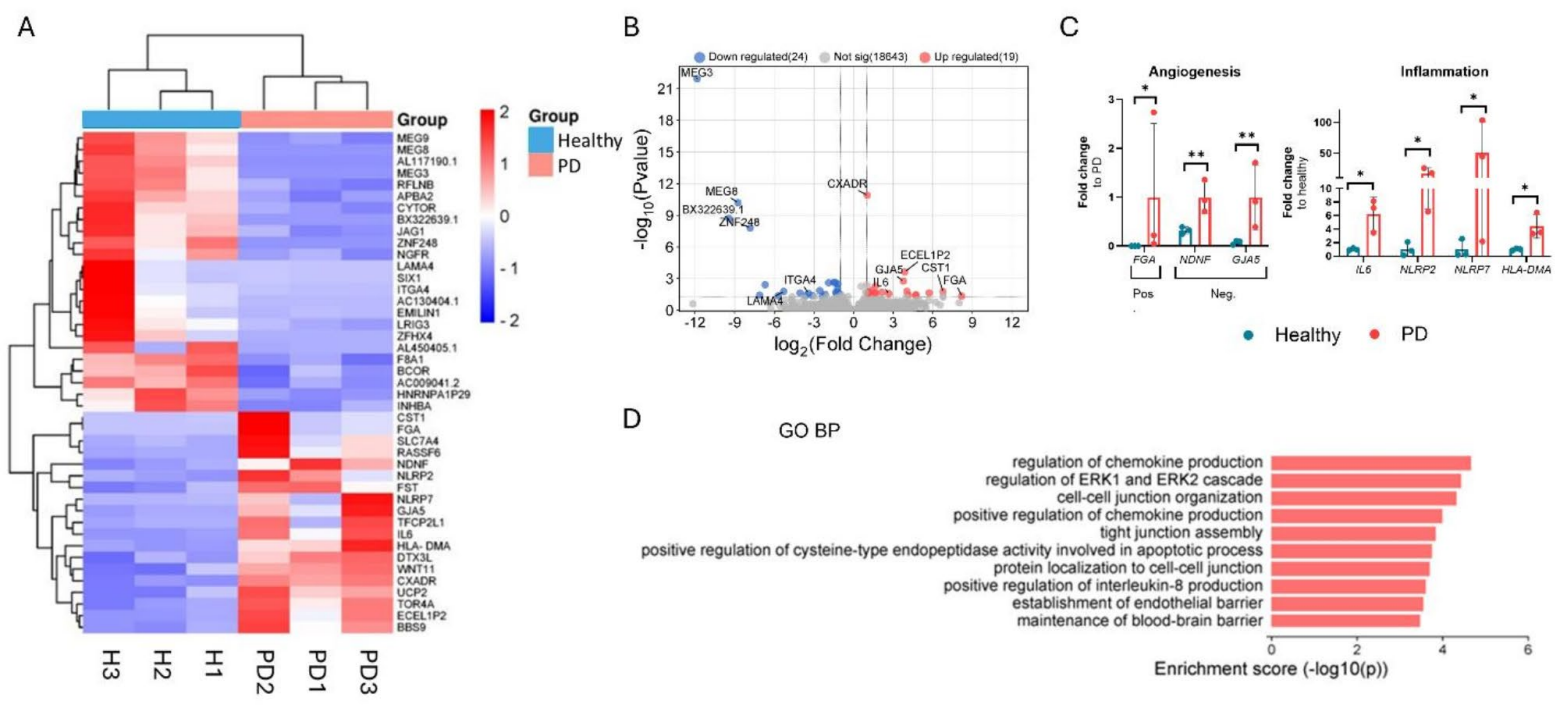
RNA expression analysis of hiPSC-derived pericyte-like cells obtained from healthy individuals and PD patients. (**A**)Cluster heatmap of significant differentially expressed genes between healthy and PD lines based on Padj value (< 0.05) and absolute log2 Fold > 1(**B**)Volcano plot of up- and downregulated DEGs between healthy and PD pericyte-like cells (**C**)Expression of genes associated with angiogenesis and inflammation. (+/-SD)(**D**)GO BP analysis of upregulated genes in PD lines compared to healthy lines. (From genes with p-value < 0.05). GO BP, gene ontology biological processes. Padj*<0.05, Padj**<0.01

To discover how the *LRRK2* G2019S mutation affects transcriptomics in pericyte-like cells, RNA-sequencing data from healthy donors and PD patients were compared. In this comparison, we identified 24 downregulated and 19 upregulated DEGs in PD pericyte-like cells (Fig. 4a, b). Among these genes were long non-coding RNAs (*MEG3* and *MEG8*), angiogenesis associated genes (*FGA*, *GJA5*, and *NDNF*) and genes associated with inflammation (*IL6*, *HLA-DMA*, *NLRP2*, and *NLRP7*) (Fig. 4c). Since only 43 DEGs were identified with an adjusted p-value < 0.05, the pathway analysis with Enrichr was made for DEGs with p-value < 0.05. Out of these DEGs, 325 genes were downregulated, and 412 genes were upregulated. The examination of gene ontology biological processes (GO BP) revealed upregulation of chemokine production, tight junction assembly, and BBB maintenance in PD lines (Fig. 4d). The KEGG pathway analysis confirmed the upregulation of tight junction associated pathways (Fig. S3).

### Altered cytokine release and migration in PD pericyte-like cells

**Fig. 5:**
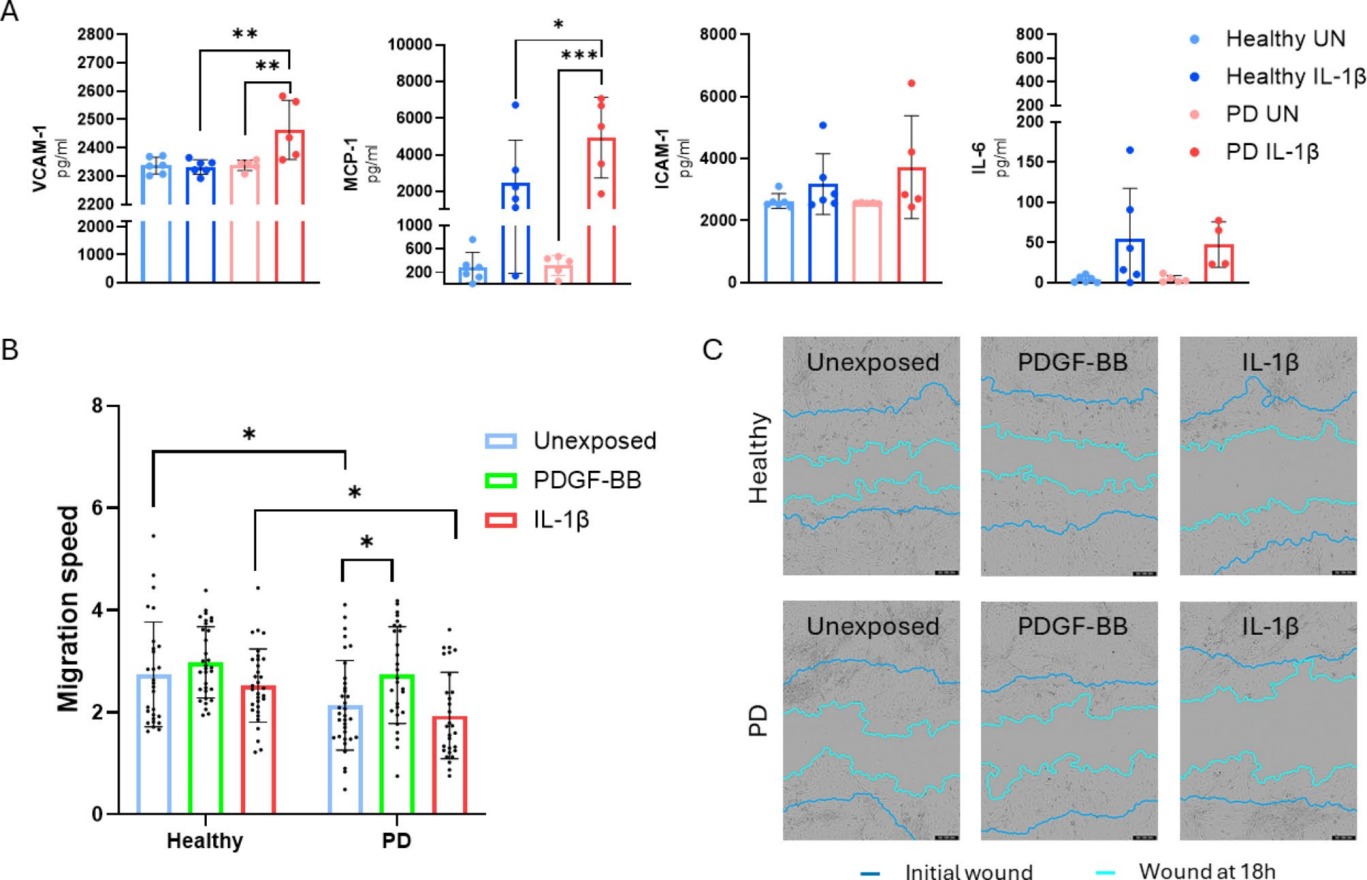
The release of inflammatory mediators and migration of hiPSC-derived pericyte-like cells. (**A**) Release of inflammatory mediators sVCAM-1, sICAM-1, MCP-1, and IL-6 with and without IL-1β exposure. (Healthy *n* = 6, PD *n* = 5) (+/-SD) One-way ANOVA with Tukey´s multiple comparison test (**B**) Changes in migration speed of hiPSC-derived pericyte-like cells calculated as a change in relative wound density %/h between timepoints 3 h and 18 h with and without PDGF-BB or IL-1β exposure. (Healthy unexposed *n* = 28, Healthy PDGF-BB *n* = 32, Healthy IL-1β *n* = 32, PD unexposed *n* = 34, PD PDGF-BB *n* = 28, PD IL-1β *n* = 29) (+/-SD) Two-way ANOVA with Bonferroni’s multiple comparison test (**C**) Representative images of migration with different exposures in healthy and PD lines. Images taken with IncuCyte. n = number of replicates, p*<0.05, p**<0.01, p***<0,001

Given the important role of pericytes in several processes, such as inflammation and angiogenesis, we conducted analysis to examine the behavior of healthy and PD pericyte-like cells in response to IL-1β. In the absence of this stimuli, there was no difference in the release of inflammatory mediators between healthy and PD lines (Fig. 5a). Nevertheless, in cells exposed to IL-1β, there was a clear trend indicating an elevated release of inflammatory mediators in both healthy and PD lines with some exceptions, such as VCAM-1. The release of soluble VCAM-1 was equivalent in both exposed and unexposed samples, but only in healthy lines, while in PD lines the release of VCAM-1 after IL-1β was significantly increased.

The migration of pericytes is strongly linked to the process of angiogenesis. Therefore, we looked at the migration of pericyte-like cells and investigated the impact of PDGF-BB or IL-1β exposures on their migration speed. PDGF-BB stimulated migration in both healthy and PD lines, even though only in PD lines the difference was significant when compared to unstimulated (Fig. 5b, c). IL-1β suppressed migration, but the changes were not significant. In addition, in unexposed and IL-1β exposed PD lines the migration speed was significantly reduced compared to healthy lines with same exposures. The differences in initial wound size might affect the results so we compared the initial wound width between healthy and PD samples and wound no significant differences in (Fig. S5). We also explored whether exposure to IL-1β affects differentially on permeability in EC co-cultures with healthy and PD pericyte-like cells, but after exposure to IL-1β, the ECs lost their monolayer formation and thus also their capability to form a barrier (Fig. S6).

## Discussion

Pericytes have historically been a neglected cell type. Although they were first found in the late 19th century, there was a lack of significant research on them for the next century [20]. The diverse population of pericytes, which can be challenging to distinguish, has impeded scientific progress on them. However, advances in technology and methodologies have facilitated the identification and examination of pericytes, leading to an exponential increase in pericyte research in the last 20 years. The findings from the in vivo and in vitro investigations [13–15] have suggested the involvement of pericytes in PD pathology though the extent of their contribution remains unclear.

The development in iPSC-technology has helped pericyte research as it provides an unlimited source of cells. The hiPSC-derived pericytes produced with the neural crest protocol from Faal et al. [17] have already been used in several studies and have been shown to possess similar properties compared to human brain vascular pericytes (HBVP) [17, 21]. In this study we produced iPSC-derived pericyte-like cells based on the same protocol, but also tested two different molecules (TGFβ3 and SB431542) for pericyte differentiation. We showed the expression of CD13, PDGFRα/β and αSMA in ICC stainings and PDGFRB, CSPG4 and VTN in RT-qPCR, which have been reported earlier in HBVPs and iPSC-derived pericytes, in our cells. We also demonstrated that PDGF-BB induced migration in pericyte-like cells, which is in line with the previous study showing PDGF-BB to induce a proliferative response in HBVPs [21] as migration and proliferation are closely related to each other [22]. Overall the pericyte-like cells used in this study express pericyte associated markers and show similar functional properties as reported earlier in HBVPs. While TGFβ3 and SB431542 generated cells were similar in terms of their morphology and the presence of pericyte markers in ICC stainings, the RNA sequencing uncovered clear differences between the cells. We observed increased expression of pericyte markers and upregulation in pericyte associated pathways in TGFβ3 differentiated cells and thus TGFβ3 was chosen for later experiments.

When comparing healthy and PD lines we observed no significant differences in cell morphology, neither did we see any significant effect on endothelial permeability. Both healthy and PD pericyte-like cells expressed LRRK2 and SNCA genes, showing no significant difference in expression levels. However, despite the expression of SNCA in the cells, α-synuclein remained absent. The absence of α-synuclein in the pericyte-like cells aligns with previous findings from in vitro cultures of primary human brain pericytes [13].

At the transcriptomic level, healthy and PD pericytes were also very similar, with only 43 differentially expressed genes. However, we observed notable differences within those genes. Prominent alterations in PD lines included the decrease in the expression of maternally expressed genes 3 (*MEG3*) and − 8 (*MEG8*) as well as the changes in genes *NDNF* and *GJA5*, which negatively regulate angiogenesis. *MEG3* and *MEG8* are long noncoding RNAs that are known for their regulative roles in cell proliferation and migration. The studies with *MEG8* have indicated cell type dependent effects on proliferation and migration. Even though multiple studies have demonstrated that *MEG8* expression enhances proliferation and migration of cancer cells [23] as well as of vascular endothelial cells [24, 25], studies with vascular smooth muscle cells showed the opposite [26, 27]. *MEG3* has been associated with PD, as several studies have identified altered levels of *MEG3* in PD patients. Specifically, two studies found lower levels of *MEG3* in plasma [28, 29] while one study reported increased levels [30]. Furthermore, there appears to be a correlation between the expression of *MEG3* and *LRRK2*, as the overexpression of *MEG3* resulted in an enhanced expression of *LRRK2* in SH-SY5Y cells exposed to MPP^+^ [29]. Currently, there are only a few studies on the functioning of *MEG3* and − *8* in pericytes. Considering our existing knowledge on *MEG3* and *MEG8*, and the role of pericytes in vascular functions, investigating the specific mechanism by which *MEG3* and *MEG8* function in pericytes and ECs could provide useful insight into vascular changes identified in PD.

The transcriptome data also indicated increased expression of genes linked to inflammation in PD pericyte-like cells. Additionally, pathway analysis revealed alterations in processes related to the regulation of chemokine production and interaction between cytokines and cytokine receptors. Under basal conditions, the release of cytokines was comparable in both healthy and PD lines. However, when the cells were exposed to IL-1β, a pro-inflammatory cytokine known to be elevated in PD [31, 32], the secretion of sVCAM-1 and MCP-1 was significantly increased in PD lines compared to healthy lines. The impact of IL-1β on primary human brain pericytes has been previously investigated [33] with the similar finding that IL-1β exposure resulted in the release of sVCAM-1 and MCP-1. Clinical studies have shown that sVCAM-1 levels in plasma of the PD patients are higher than those of healthy individuals [34]. These levels are also correlated with disease progression, particularly with motor impairment [34]. In regards of MCP-1, there have been contradictory results about whether MCP-1 levels are elevated in PD. At least one study reported increased MCP-1 levels in cerebrospinal fluid of PD patients [35] while another study found no difference compared to healthy controls but suggested that the levels of MCP-1 might correlate with disease progression and motor dysfunction [36]. sVCAM-1 is able to disrupt brain endothelial integrity and is also associated with tumor angiogenesis. On the other hand, MCP-1 plays a crucial role in inflammation by attracting inflammatory cells and enhancing the production of other inflammatory factors. Therefore, elevated amounts of sVCAM-1 and MCP-1 secreted by PD pericytes could result in heightened permeability of the BBB and an intensified inflammatory response. However, due to the total loss of barrier property in ECs exposed to IL-1β, we were unable to determine whether exposure to IL-1β would cause a different response in permeability in EC co-cultures with PD and healthy pericyte-like cells. Our results suggest that hiPSC-derived pericyte-like cells carrying the *LRRK2* G2019S mutation exhibit a more reactive phenotype in pericytes. Similar changes in reactivity due to the *LRRK2* G2019S mutation have been previously reported in glial cells, astrocytes and microglia, which are likewise activated during neuroinflammation [37–39]. Yet, the impact of this mutation on pericytes has not been studied previously.

Pericyte migration and angiogenesis are closely related to each other, as the migration of pericytes is essential for the proper formation of new blood vessels. Prior research suggests that during the initial stages of angiogenesis, migration of pericytes from the vessel is necessary to initiate ECs sprouting and later to stabilize the newly formed vessels [22]. In PD there is evidence of pathological angiogenesis from postmortem studies [7, 8] as well as animal models [40], but the reasons behind it remains mostly unknown. Even though in our studies we demonstrated compromised migration in unexposed pericytes with LRRK2 mutation, the pro-angiogenic stimuli had stronger effect on PD pericytes. Thus, it is possible that pro-angiogenic environment in PD brain induces pericytes affecting the angiogenesis. However, further studies are needed to ascertain their effect on vascular changes in PD.

### Limitation of our study.

Pericyte migration is important for normal angiogenesis. Hampered migration can alter angiogenesis and destabilizing of newly formed blood vessels. In this research, we examined only one pro-angiogenic molecule, but it would be advantageous to additionally investigate, for example, VEGF. VEGF is elevated in the brains of the PD patients and it could be examined if different concentrations lead to changes in pericyte-like cells between healthy and PD lines. We also did not test how cells would behave when exposed simultaneously to pro-inflammatory and pro-angiogenic stimuli. The study from Kang et al. has demonstrated that when pericytes are present, the antiangiogenic effect of TNFα on ECs can be turned into a pro-angiogenic effect, in the presence of the pro-angiogenic molecule VEGF [11]. Thus, it is possible that combined exposure with PDGF-BB and IL-1β may have a distinct consequence on migration than just individual exposures with PDGF-BB and IL-1β.

In addition, we were not able to include an isogenic line in which the *LRRK2* G2019S mutation has been corrected. The isogenic line with otherwise similar genome to PD line except for the LRRK2 gene would have allowed us to determine if the changes we see in the PD pericyte-like cells are specifically due to LRRK2 G2019S mutation. However, without isogenic lines we cannot exclude the possibility of other genetic factors contributing to these changes.

## Conclusions

In summary, pericyte-like cells with LRRK2 G2019S mutation show moderate alterations in the transcriptome profile and changes in migration under basal conditions. They also possess a more reactive phenotype characterized by an increased release of inflammatory mediators upon exposure to pro-inflammatory stimuli. In addition, changes seen in PD-pericyte-like cells including altered levels of MEG3 and VCAM-1 have also been reported in PD patients.

## Electronic supplementary material

Below is the link to the electronic supplementary material.

Supplementary Material 1

## Data Availability

The transcriptomics data supporting the findings of this study are available from the corresponding author upon reasonable request through Zenodo. (Healthy lines differentiated with SB431542 doi: 10.5281/zenodo.13734563 (https://zenodo.org/records/13734563), Healthy lines differentiated with TGFb3 doi: 10.5281/zenodo.13735656 (https://zenodo.org/records/13735656), PD lines differentiated with TGFb3 doi: 10.5281/zenodo.13735939 (https://zenodo.org/records/13735939).

## Abbreviations

BBB: Blood brain barrier
BMEC: Brain microvessel endothelial cell
CNS: Central nervous system
CBA: Cytometric bead array
DEG: Differentially expressed gene
EC: Endothelial cell
GO: BP Gene ontology biological processes
HBVP: Human brain vascular pericytes
hiPSC: Human induced pluripotent stem cell
KEGG: Kyoto Encyclopedia of genes and genomes
Papp: Apparent permeability
PD: Parkinson’s disease
