# Supplementary material for: Mutated LRRK2 induces a reactive phenotype and alters migration in human iPSC-derived pericyte-like cells
Source: Fluids Barriers CNS. 2024 Nov 18;21:92. doi: 10.1186/s12987-024-00592-y (PMC11571670; doi:10.1186/s12987-024-00592-y)
Supplement: Supplementary file 1 — Supplementary Material 1 [file 12987_2024_592_MOESM1_ESM.docx]

# Supplementary materials

*Supplementary table S1.* ***List of antibodies used in immunocytochemistry***

| **Type** | **Antibody** | **Origin** | **Manufacturer** | **Cat. No.** | **Dilution** | **Fixation** |
| --- | --- | --- | --- | --- | --- | --- |
| Primary | aSMA | mouse | Sigma | A5228 | 1:300 | FA/MeOH |
|  | CD13 | mouse | Santa Cruz | sc-166105 | 1:200 | FA |
|  | PDGFRa&b | rabbit | Abcam | ab32570 | 1:100-125 | FA/MeOH |
|  | VE-cadherin | mouse | Santa Cruz | sc-9989 | 1:100 | MeOH |
| Secondary | anti-mouse 488 | goat | Invitrogen | A11001 | 1:300 |  |
|  | anti-rabbit 568 | goat | Invitrogen | A11011 | 1:300 |  |

*Supplementary table S2.* ***List of primers used in RT-qPCR***

| **Gene symbol** | **Gene name** | **TagMan Gene expression assay ID** |
| --- | --- | --- |
| ACTB | Beta-actin | 4326315 E |
| CSPG4 | Chondroitin Sulfate Proteoglycan 4 (Neural/glial antigen 2, NG2) | Hs00426981_m1 |
| LRRK2 | Leucine rich repeat kinase 2 | Hs00968209_m1 |
| OCLN | Occludin | Hs00170162_m1 |
| PDGFRb | Platelet derived growth factor beta | Hs01019589_m1 |
| SNCA | Synuclein alpha | Hs01103383_m1 |
| VTN | Vitronectin | Hs00169863_m1 |

*
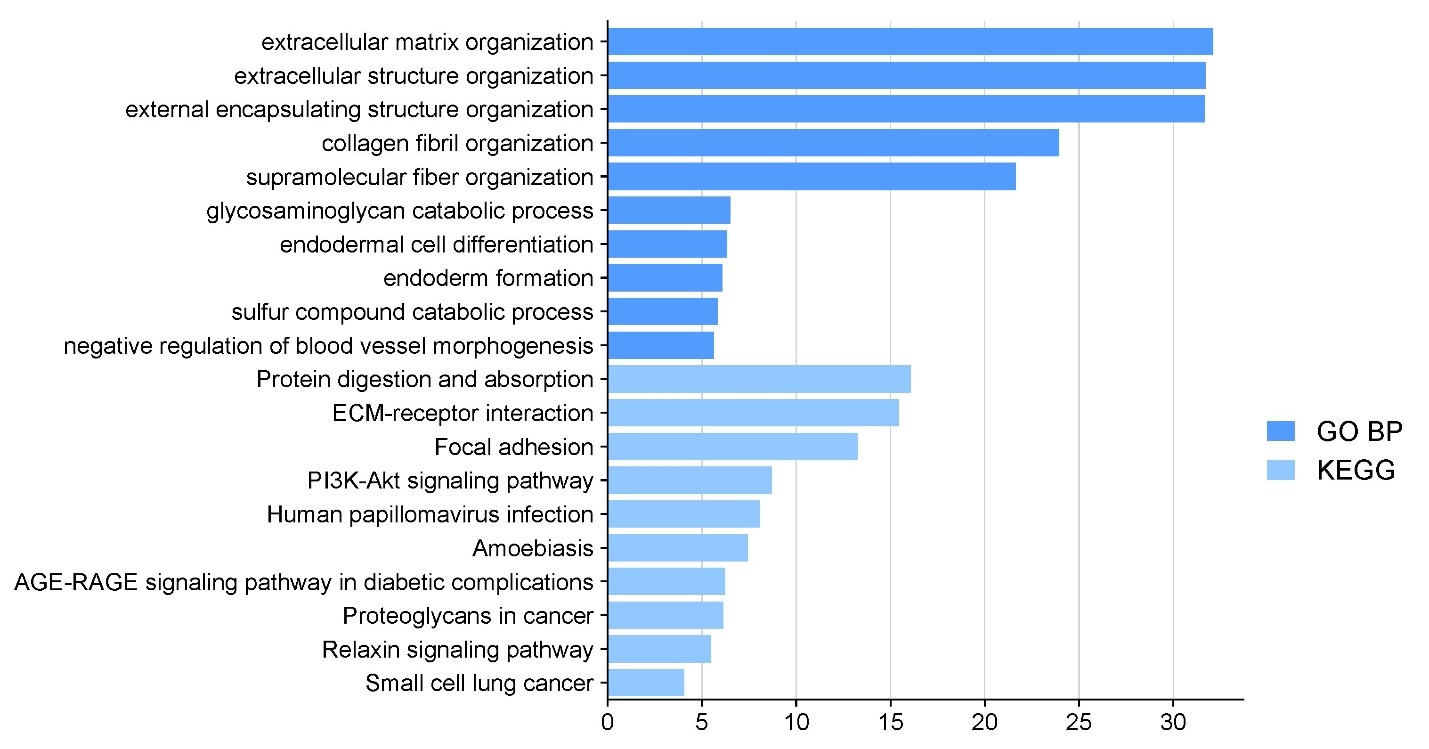

Supplementary figure S1:* ***Pericyte associated pathways.*** *GO BP and KEGG pathways associated with pericytes. Based on 98 pericyte genes from Pericyte: brain CellMarker_Augmented_2021 data set in EnrichR.*


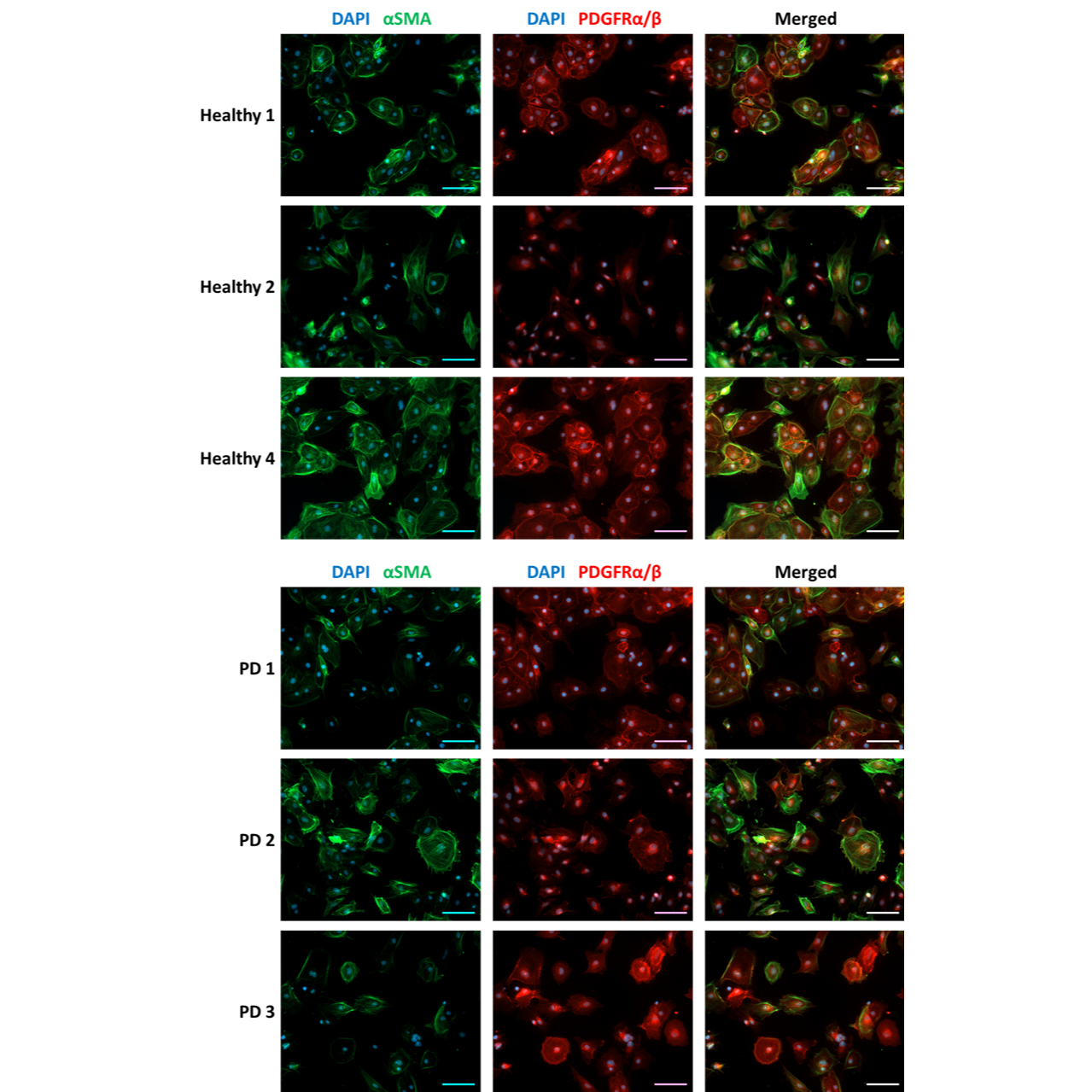


*Supplementary figure S2:* ***Expression of pericyte markers in hiPSC-derived pericyte-like cells.*** *Representative immunofluorescence images of healthy (H1,2 and 4) and PD (PD1-3) pericyte-like cells stained for αSMA and PDGFRβ/α. Scale bar 100 µm.*


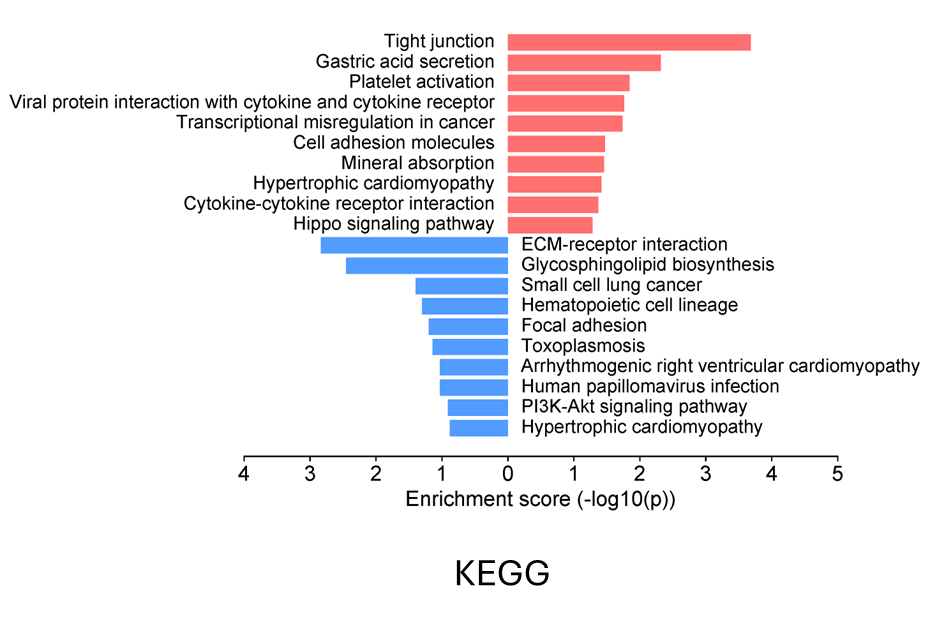
*Supplementary figure S3:* ***KEGG pathway analysis of up- and down regulated genes in PD pericyte-like cells compared to healthy cells.*** *(From genes with p-value <0.05).*

*
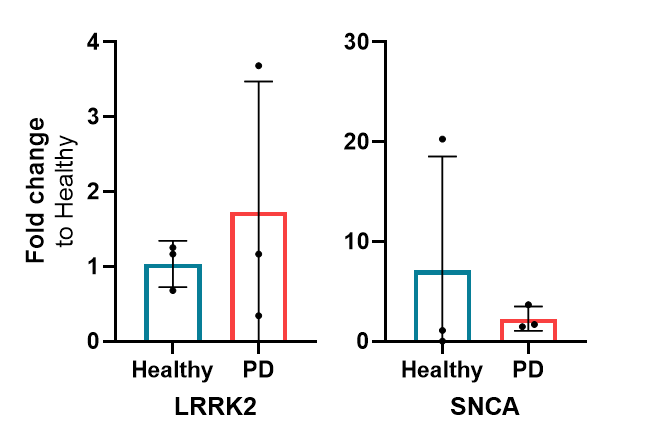

Supplementary figure S4:* ***Relative expression of LRRK2 and SNCA in healthy and PD pericyte-like cells.*** *(n=3) (+/-SD) Unpaired t-test.*

*
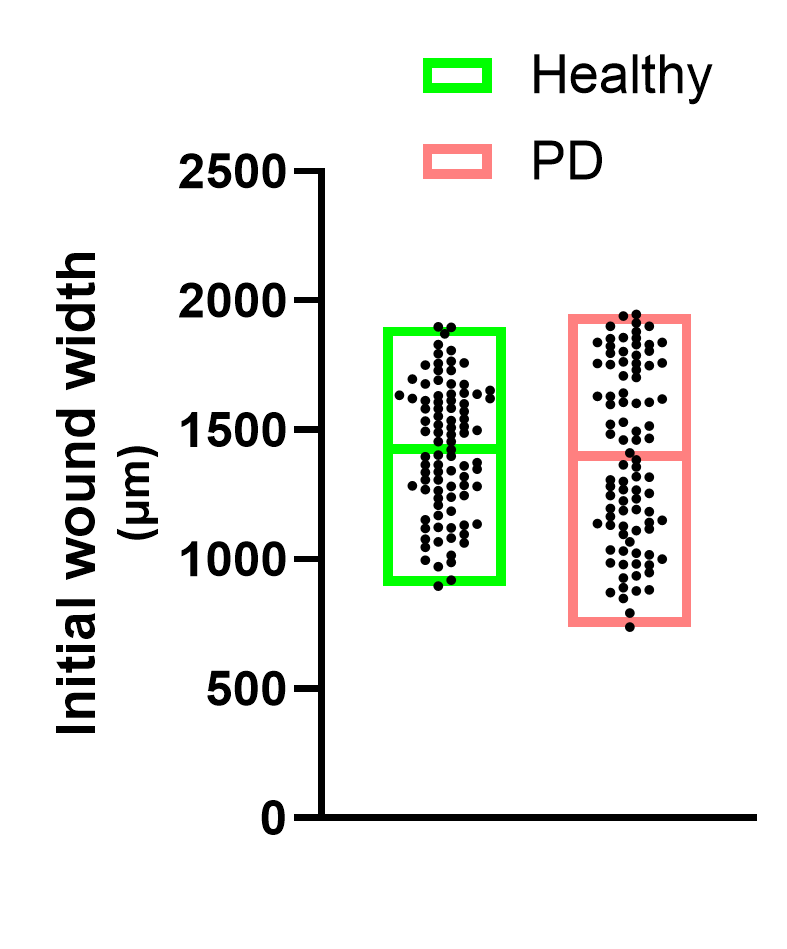
*

*Supplementary figure S5:* ***Initial wound width in healthy and PD samples in scratch wound assay.*** *Unpaired t-test*

*
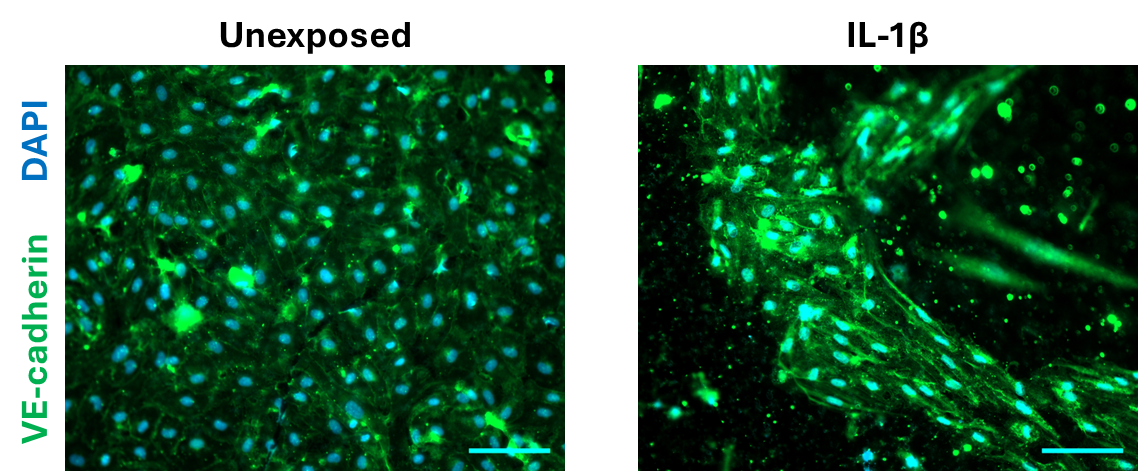

Supplementary figure S5:* ***Representative images of unexposed and IL-1β exposed endothelial cells on inserts stained with VE-cadherin.*** *Scale bar 100 µm.*
